# Evaluation of thresholding methods for the quantification of [^68^Ga]Ga-PSMA-11 PET molecular tumor volume and their effect on survival prediction in patients with advanced prostate cancer undergoing [^177^Lu]Lu-PSMA-617 radioligand therapy

**DOI:** 10.1007/s00259-023-06163-x

**Published:** 2023-03-02

**Authors:** Moon Kim, Robert Seifert, Jana Fragemann, David Kersting, Jacob Murray, Frederic Jonske, Kelsey L. Pomykala, Jan Egger, Wolfgang P. Fendler, Ken Herrmann, Jens Kleesiek

**Affiliations:** 1 Institute for Artificial Intelligence in Medicine (IKIM), University Hospital Essen, Essen, Germany; 2 Department of Diagnostic and Interventional Radiology and Neuroradiology, University Hospital Essen, Essen, Germany; 3 Department of Nuclear Medicine, University Hospital Essen, Essen, Germany; 4 German Cancer Consortium (DKTK), University Hospital Essen, Essen, Germany; 5 Cancer Research Center Cologne Essen (CCCE), Essen, Germany

**Keywords:** PSMA PET/CT, Prostate cancer, Thresholding, Image biomarker, Tumor volume

## Abstract

**Purpose:**

The aim of this study was to systematically evaluate the effect of thresholding algorithms used in computer vision for the quantification of prostate-specific membrane antigen positron emission tomography (PET) derived tumor volume (PSMA-TV) in patients with advanced prostate cancer. The results were validated with respect to the prognostication of overall survival in patients with advanced-stage prostate cancer.

**Materials and methods:**

A total of 78 patients who underwent [^177^Lu]Lu-PSMA-617 radionuclide therapy from January 2018 to December 2020 were retrospectively included in this study. [^68^Ga]Ga-PSMA-11 PET images, acquired prior to radionuclide therapy, were used for the analysis of thresholding algorithms. All PET images were first analyzed semi-automatically using a pre-evaluated, proprietary software solution as the baseline method. Subsequently, five histogram-based thresholding methods and two local adaptive thresholding methods that are well established in computer vision were applied to quantify molecular tumor volume. The resulting whole-body molecular tumor volumes were validated with respect to the prognostication of overall patient survival as well as their statistical correlation to the baseline methods and their performance on standardized phantom scans.

**Results:**

The whole-body PSMA-TVs, quantified using different thresholding methods, demonstrate a high positive correlation with the baseline methods. We observed the highest correlation with generalized histogram thresholding (GHT) (Pearson *r* (*r*), *p* value (*p*): *r* = 0.977, *p* < 0.001) and Sauvola thresholding (*r* = 0.974, *p* < 0.001) and the lowest correlation with Multiotsu (*r* = 0.877, *p* < 0.001) and Yen thresholding methods (*r* = 0.878, *p* < 0.001). The median survival time of all patients was 9.87 months (95% CI [9.3 to 10.13]). Stratification by median whole-body PSMA-TV resulted in a median survival time from 11.8 to 13.5 months for the patient group with lower tumor burden and 6.5 to 6.6 months for the patient group with higher tumor burden. The patient group with lower tumor burden had significantly higher probability of survival (*p* < 0.00625) in eight out of nine thresholding methods (Fig. 2); those methods were SUVmax50 (*p* = 0.0038), SUV ≥3 (*p* = 0.0034), Multiotsu (*p* = 0.0015), Yen (*p* = 0.0015), Niblack (*p* = 0.001), Sauvola (*p* = 0.0001), Otsu (*p* = 0.0053), and Li thresholding (*p* = 0.0053).

**Conclusion:**

Thresholding methods commonly used in computer vision are promising tools for the semiautomatic quantification of whole-body PSMA-TV in [^68^Ga]Ga-PSMA-11-PET. The proposed algorithm-driven thresholding strategy is less arbitrary and less prone to biases than thresholding with predefined values, potentially improving the application of whole-body PSMA-TV as an imaging biomarker.

## Introduction

Prostate cancer is the second most prevalent cancer in men and accounts for nearly 7% of cancer-related deaths in men worldwide [1]. Thus, accurate diagnosis, staging, and treatment planning of prostate cancer are of high importance. Upregulation of prostate-specific membrane antigen (PSMA) is present in over 90% of prostate cancer patients [2] and can be an early indicator for the presence of a hormone-sensitive or castrate-resistant prostate cancer phenotype [3]. PSMA-targeted positron emission tomography (PET) is, therefore, an excellent staging and restaging tool for patients with prostate cancer [4]. It has been shown that PSMA PET has a high specificity and positive predictive value in the detection of prostate cancer lesions in patients referred for the detection of intraprostatic prostate cancer, initial staging of high-risk prostate cancer, and for staging in biochemical recurrence [5–7]. Moreover, a meta-analysis of recent studies has confirmed the excellent sensitivity and specificity of PSMA PET in detecting prostate cancer and its high impact on patient management and clinical decision-making, especially in biochemical recurrence [8]. PSMA-PET-derived tumor volume (PSMA-TV) is an emerging imaging biomarker, with several studies proposing benefits for therapy response assessment [9–11] and for the prognostication of overall survival [10, 12]. The delineation of cancer foci and quantification of PSMA-TV is highly dependent on thresholding methods. The choice of the respective thresholding method can have a large influence on the estimated tumor volume and, therefore, cause a significant bias. The European Association of Nuclear Medicine (EANM) recommends the use of percentage-based thresholding (PBT) of maximal standardized uptake value (SUVmax) of the delineated tumor foci for assessment of the molecular tumor volume in [^18^F]F-fluordesoxyglucose (FDG) PET [13]. For PSMA PET, no such recommendation exists and various approaches have been applied. Currently, PBT with iso-contours set at 41% [14], 45% [9, 14], and 50% [12, 14, 15] of SUVmax within the respective tumor focus and fixed thresholding of the standardized uptake value (i.e., SUV ≥ 3) are used most widely. The latter was validated in prospective trials, e.g., TheraP [16] and Vision [17]. The application of percentage-based or fixed thresholds on spatially heterogeneous in vivo tumor foci with varying forms, sizes, localizations, and body locations (e.g., soft tissues and bones) is not ideal. This may lead to over- or underestimation of PSMA-TV. In the case of PBT, it may systematically underestimate the molecular tumor volume when high focal tracer uptake with lower uptake of the surrounding tumor is present. Overestimation may occur after performing PSMA-targeted therapy, where the measured tumor volume can paradoxically increase due to decreased SUVmax in response to therapy [12]. Furthermore, experiments with modern 3D-printed irregularly formed phantoms with heterogeneous uptake suggest that adaptive thresholding may be a more sophisticated approach to overcome the limitations of fixed or PBT values [18].

The aim of this study was to systematically evaluate the effects of different thresholding algorithms that are well established in computer vision on the quantification of PSMA-TV and to validate the results with respect to the prognostication of overall survival in patients with advanced prostate cancer. Moreover, phantom images were analyzed to validate the results in an investigation of lesions with pre-defined volumes.

## Materials and methods

### Patients and preparation

At Essen University Hospital, patients with advanced prostate cancer who show disease progression despite chemotherapy are considered for [^177^Lu]Lu-PSMA-617 therapy. Assessment for this treatment includes a pre-therapy PSMA-PET/CT examination with increased uptake of PSMA ligands and verification of good renal and bone marrow function. These patients have high tumor burden and are followed up over an extended period at our site, thus providing valid clinical data for the survival analysis. In total, 78 patients were retrospectively included in this study. Patients with advanced prostate cancer who received [^177^Lu]Lu-PSMA-617 radioligand therapy (RLT) from January 2018 to December 2020 were initially identified in the local database. The inclusion criteria were histopathologically proven adenocarcinoma of the prostate and the presence of an on-site [^68^Ga]Ga-PSMA-11 PET/CT examination before administration of the first cycle of [^177^Lu]Lu-PSMA-617 radionuclide therapy. PSA values were obtained within 30 days of the diagnostic PET/CT examination in an outpatient setting and before the first [^177^Lu]Lu-PSMA617 radionuclide therapy. At the time of PET/CT imaging, all patients had advanced prostate cancer (UICC stage IV). Overall survival (OS) was defined as the time from the first cycle of RLT until death or last follow-up. Detailed patient characteristics are listed in Table 1. The study was approved by the institutional review board and local ethics committee (Ethics committee, University Duisburg-Essen, Faculty of Medicine, Ethics protocol number 19-8570-BO). The patients gave written informed consent for the clinical examination.

**Table 1:** Patient characteristics

Characteristic	Mean (SD)	*n* (%)
Total number of patients		78 (100)
Age (years)	71.2 (8.0)	
Weight (kg)	85.6 (17.9)	
Gleason score		
6		5 (6.4)
7a		4 (5.1)
7b		6 (7.7)
8		30 (38.5)
9		24 (30.8)
10		9 (11.5)
UICC stage		
IV		78 (100)
PSA (ng/ml)	314.1 (504.3)	
Site of metastases		
Liver metastases		12 (15.4)
Bone metastases		70 (89.7)
Lymph node metastases		49 (62.8)
Lung metastases		8 (10.3)

### PET acquisition

PET/CT data were acquired using a Biograph Vision 600 and a Biograph mCT PET/CT system (Siemens Healthineers, Erlangen, Germany) according to our clinical standard PET protocols for whole-body PSMA PET/CT imaging of prostate cancer patients. PET/CT data were acquired in a prone position with arms placed overhead. PSMA-11 precursors were supplied by ABX (ABX GmbH). The intravenously administered activity of [^68^Ga]Ga-PSMA-11 was adapted to the patient’s body weight (2 MBq/kg). The mean delay between injection and PET acquisition was 72 min. CT scans were acquired immediately before the PET acquisition and used for attenuation correction.

### Validation of the thresholding methods using phantom scans

To validate the patient scan results, PET images from a previously published phantom study were re-evaluated [19]. These contain homogeneous hot lesions of a defined volume in a warm background (signal-to-background ratio 20:1). The phantom is an abdominal torso NEMA phantom (Data Spectrum, Corporation, Durham, USA) with six glass spheres filled with [^68^Ga]Ga in an aqueous solution (diameters of 6.5, 9.5, 12.6, 17.4, 22.2, and 28.0 mm). The phantom is well established to characterize the detectability and lesion quantification accuracy of PET systems. Sphere sizes, activity concentrations, and signal-to-background ratio were derived from clinical [^68^Ga]Ga-PSMA PET/CT scans to mimic prostate cancer lesions as described in a previous work [19]. PET images were acquired according to our institution’s standard PET protocol at varying acquisition times (30, 60, 90, 120, 150, 180, 210, 240, 270, 300, and 600 s), resulting in 11 measurements for each spherical insert and used for internal validation.

### Semi-automatic quantification of molecular tumor volume

**Fig. 1:**
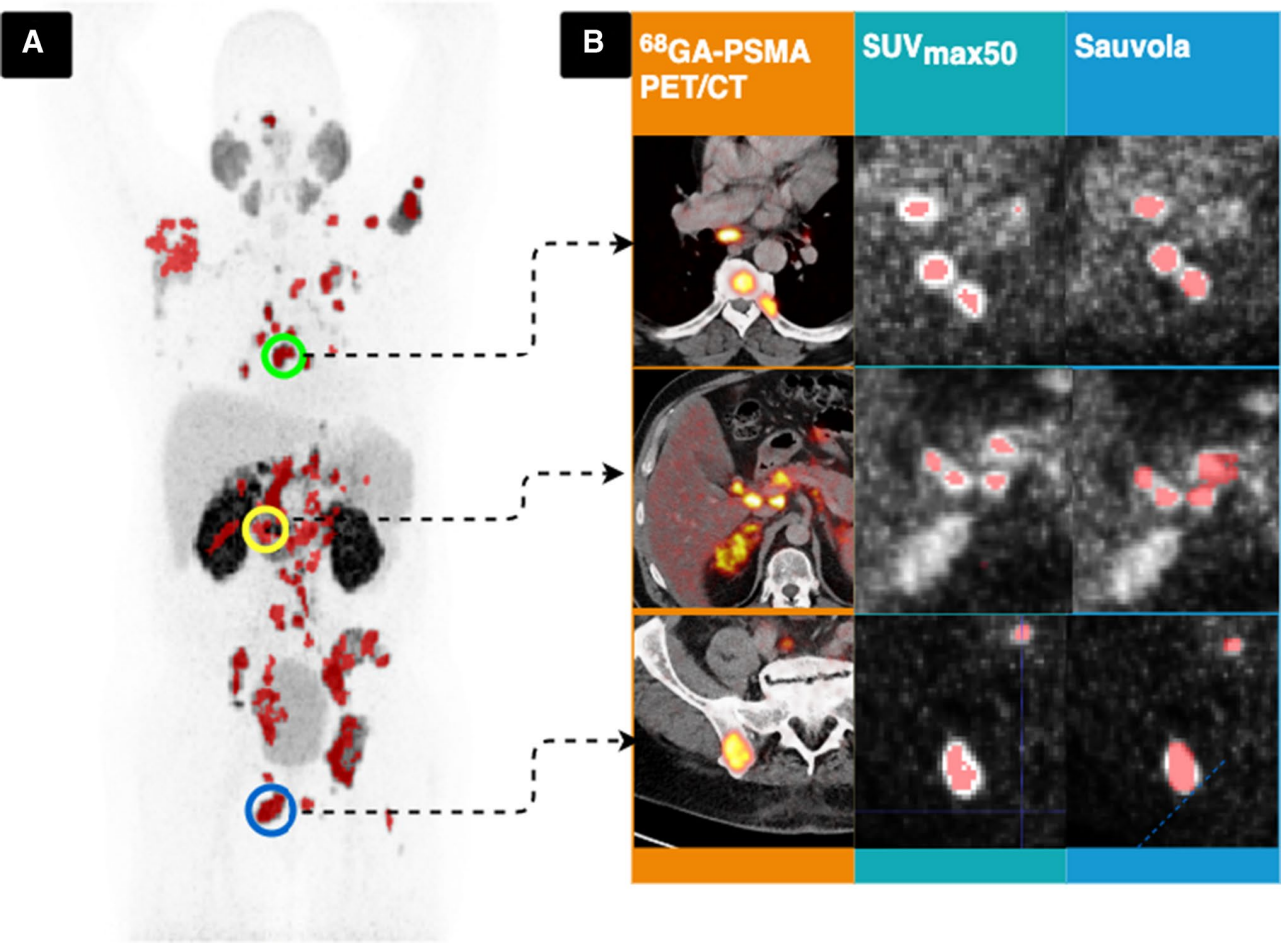
A, Maximum intensity projection of PSMA PET with overlaid thresholding binary mask of SUVmax50 for visual control. Green circle: subcarinal lymph node metastasis in the mediastinum and bone metastases in thoracic vertebrae and ribs; yellow circle: diffuse intra-abdominal lymph node metastasis at the hepatic hilum; blue circle: bone metastasis in the right ilium. B, Example comparison of PSMA PET positive lesions using the binary mask of SUVmax50 and Sauvola thresholding method

All PET images were first analyzed semi-automatically using the MI Whole Body Analysis Suite (MIWBAS, version 1.0; Siemens Medical Solution USA, Inc., Knoxville, TN). We followed the protocol outlined in a previous study [12]. First, thresholding based on liver background uptake value was applied as in qPSMA [20], then physiological tracer uptakes were manually excluded. A centrosymmetric 3-way structuring matrix was used to identify coherent tumor foci. Voxel clusters with a volume of less than 0.5 ml were discarded according to the protocol. Next, voxel clusters with increased tracer uptake were manually classified as a tumor focus or were dismissed as physiological accumulation by two independent nuclear medicine physicians (3 and 4 years of experience). The resulting image was used to create a binary exclusion mask of false-positive and physiological uptake of PET images. Percentage-based thresholding at 50% of SUVmax (SUVmax50) was applied to the images with voxel clusters to quantify the baseline PSMA-TV of each lesion where physiological tracer uptakes were absent [12, 20]. Fixed thresholding at SUV ≥3 and algorithms from computer vision were directly applied to the original PET images followed by the removal of physiological and false-positive uptakes using the previously generated binary exclusion masks. Regarding the phantom images, the algorithms were directly applied. The applied algorithms from computer vision were generalized histogram thresholding (GHT) [21], Otsu’s thresholding [22], modified Otsu thresholding for multiple threshold classes [23], Yen automatic multilevel thresholding [24], minimum cross-entropy thresholding [25], Niblack thresholding [26], and Sauvola thresholding [27]. The computations of thresholding algorithms were performed by means of image processing using the Python language with the numpy, pandas, scipy, and sklearn packages (Python Software Foundation; Python Language Reference, version 3.8.2). Checkpoints were defined in the automatic image processing pipeline, where maximum intensity projections of the PET with overlaid binary masks of the thresholding methods were saved for visual inspection (Fig. 1).

### Systematic evaluation of thresholding methods

For the evaluation of thresholding methods, histogram-based and local adaptive algorithms were tested, which were compared to the baseline methods (fixed thresholding of SUV ≥3 and PBT >50% of SUVmax):

Histogram-based thresholding (HBT) is a ubiquitous tool used for the binarization of images and calculates one optimal threshold value for the entire image based on the image intensity histogram [21, 22]. Preliminary testing has shown that global thresholding is not well suited for binarization of an entire PET volume image on account of the low signal-to-noise ratio and high voxel intensities of physiological tracer accumulation in the urinary tract. Therefore, histogram-based thresholding methods were locally applied to the PET images as outlined in a previous study [12].

Local adaptive thresholding (LAT) works with an entirely different strategy for image binarization. It aims to calculate a thresholding value for each voxel within a spherical or rectangular area with its respective predetermined radius or window size [26, 27]. Therefore, LAT was first applied to the whole PET image to calculate a thresholding image. Then, each voxel intensity in the PET image was compared to the threshold image for binarization (threshold image > PET image).

The characteristics of the thresholding methods used in this study are summarized in Table 2. Initialization parameters were left at default values whenever possible to reduce the chance of bias toward a particular thresholding method. The equally applied window size of 15 voxels for LAT methods was determined through empirical tests.

**Table 2:** Characteristics of the applied thresholding methods

Methods	Denoted as	Type	Initial parameters
Fixed threshold of SUV ≥3	*SUV ≥ 3*	Fixed SUV	–
Voxels >50% of SUVmax [4, 12]	*SUVmax50*	Local 50% of SUVmax	–
Generalized histogram thresholding [21]	*GHT*	Histogram based	omega = 0.5
Otsu thresholding from gray image histogram [22]	*Otsu*	Histogram based	–
Modified Otsu thresholding for multiple threshold classes [23]	*Multiotsu*	Histogram based	Higher threshold value of 3 classes
Yen automatic multilevel thresholding [24]	*Yen*	Histogram based	–
Minimum cross-entropy thresholding [25]	*Li*	Histogram based	–
Niblack local adaptive thresholding [26]	*Niblack*	Local adaptive	Window size = 15, *k* = 0.2
Sauvola local adaptive thresholding [27]	*Sauvola*	Local adaptive	Window size = 15, *k* = 0.2

### Statistical analysis

Statistical analysis was performed using Python language (version 3.8.2). The whole-body PSMA-TVs are presented as mean values with ± 1 standard deviation. The scipy Python package (version 1.6.2) was used for the Pearson correlation analysis. Survival data were analyzed using the lifelines package (version 0.26.3) (https://lifelines.readthedocs.io/), including Kaplan–Meier, log-rank test, and Cox regression. Kaplan–Meier and log-rank tests were conducted on stratified PSMA-TV by median and quartiles. Multivariate Cox’s proportional hazards regression analysis was performed with age and weight as static covariates. The assumption of proportional hazard was assessed visually by inspecting the log-log plots. Plots were generated using matplotlib (version 3.3.4) and seaborn (version 0.11.2) Python packages. For the phantom scan, the agreement between the volume estimated from threshold methods and reference volume from spherical inserts was analyzed using Bland–Altman plots, whereas ±2 standard deviations indicate the limits of agreement. *P* values are rated as statistically significant with a value of less than 0.05 divided by the number of tests performed according to Bonferroni to reduce the chance of false positives.

## Results

### Whole-body PSMA-TV obtained via different thresholding algorithms compared and correlated to SUVmax50 and SUV ≥3

**Fig. 2:**
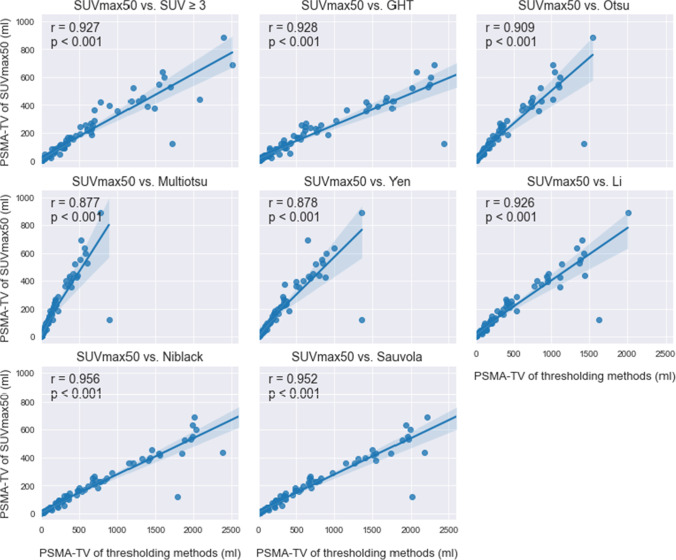
Correlation between PSMA-TV obtained via different thresholding algorithms and baseline PSMA-TV obtained via thresholding at 50% of SUVmax (*r* = Pearson *r*; *p* = *p* value)

**Fig. 3:**
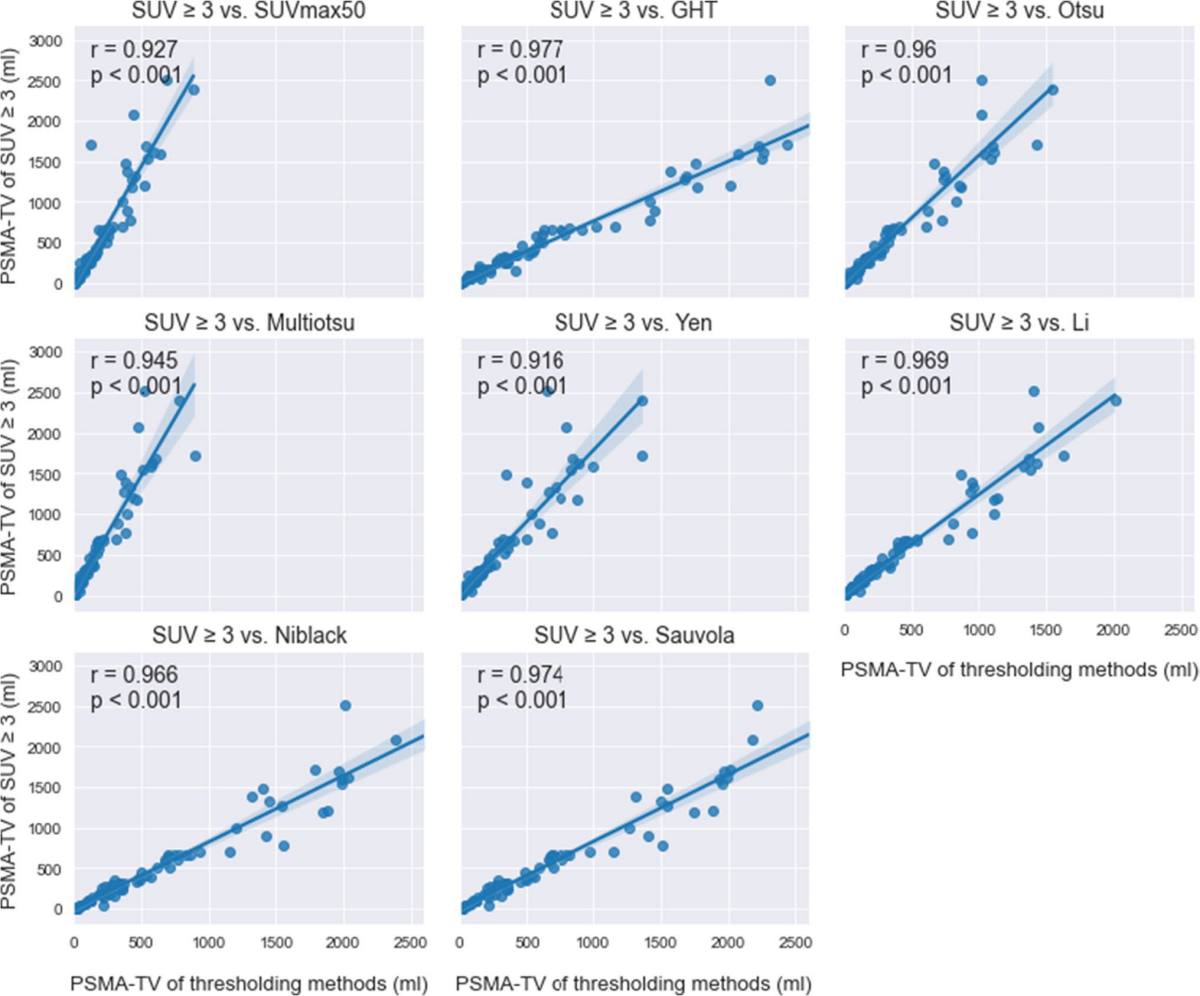
Correlation between PSMA-TV obtained via different thresholding algorithms and baseline PSMA-TV obtained via thresholding at SUV ≥ 3 (*r* = Pearson *r*; *p* = *p* value)

The whole-body PSMA-TVs, quantified using different thresholding methods, showed a high positive correlation with the baseline SUVmax50 and SUV ≥3 methods (Figs. 2 and 3). For SUVmax50, the whole-body PSMA-TVs of different thresholding algorithms were larger overall in comparison except for when compared to Multiotsu thresholding (mean PSMA-TV ± SD, Pearson *r* (*r*), *p* value (*p*): 172.67 ± 197.63 ml, *r* = 0.877, *p* < 0.001). The highest correlation of whole-body PSMA-TV to SUVmax50 was seen with Niblack (648.94 ± 703.94 ml, *r* = 0.956, *p* < 0.001), Sauvola (472.14 ± 506.27 ml, *r* = 0.952, *p* < 0.001), GHT (693.21 ± 791.83 ml, *r* = 0.928, *p* < 0.001), and Li thresholding (420.48 ± 475.98 ml, *r* = 0.926, *p* < 0.001). The lowest correlation was observed with Multiotsu (172.67 ± 197.63 ml, *r* = 0.877, *p* < 0.001) and Yen thresholding methods (285.1 ± 314.21 ml, *r* = 0.878, *p* < 0.001). The extreme outliers were a result of advanced disease with disseminated bone and liver metastases, in which the focal SUVmax partly reached 18.4, thus leaving SUV up to 9.2 not being counted toward PSMA-TV and underestimating the tumor burden in the SUVmax50 method.

Noticeably, liver metastases posed the most challenging tracer uptake for quantification of PSMA-TV, which partly explains the outliers in the correlation graph. The algorithm struggled in 3 out of 12 cases with liver metastases, especially when multiple liver metastases with slightly increased tracer uptake above physiological liver uptake value were present. It led either to overestimation with physiological liver uptake being delineated as tumor or underestimation when the algorithms failed to delineate the liver metastases (Figs. 2 and 3).

The applied thresholding algorithms had an overall higher correlation to the SUV ≥3 than to the SUVmax50 method (Figs. 2 and 3). The correlation of whole-body PSMA-TV to SUV ≥3 was in descending order: GHT (Pearson *r* (*r*), *p* value (*p*): *r* = 0.977, *p* < 0.001), Sauvola (*r* = 0.974, *p* < 0.001), Li (*r* = 0.969, *p* < 0.001), Niblack (*r* = 0.966, *p* < 0.001), Otsu (*r* = 0.96, *p* < 0.001), Multiotsu (*r* = 0.945, *p* < 0.001), and Yen (*r* = 0.916, *p* < 0.001).

### Stratification of whole-body PSMA-TV obtained with thresholding methods allows for prediction of overall survival

**Fig. 4:**
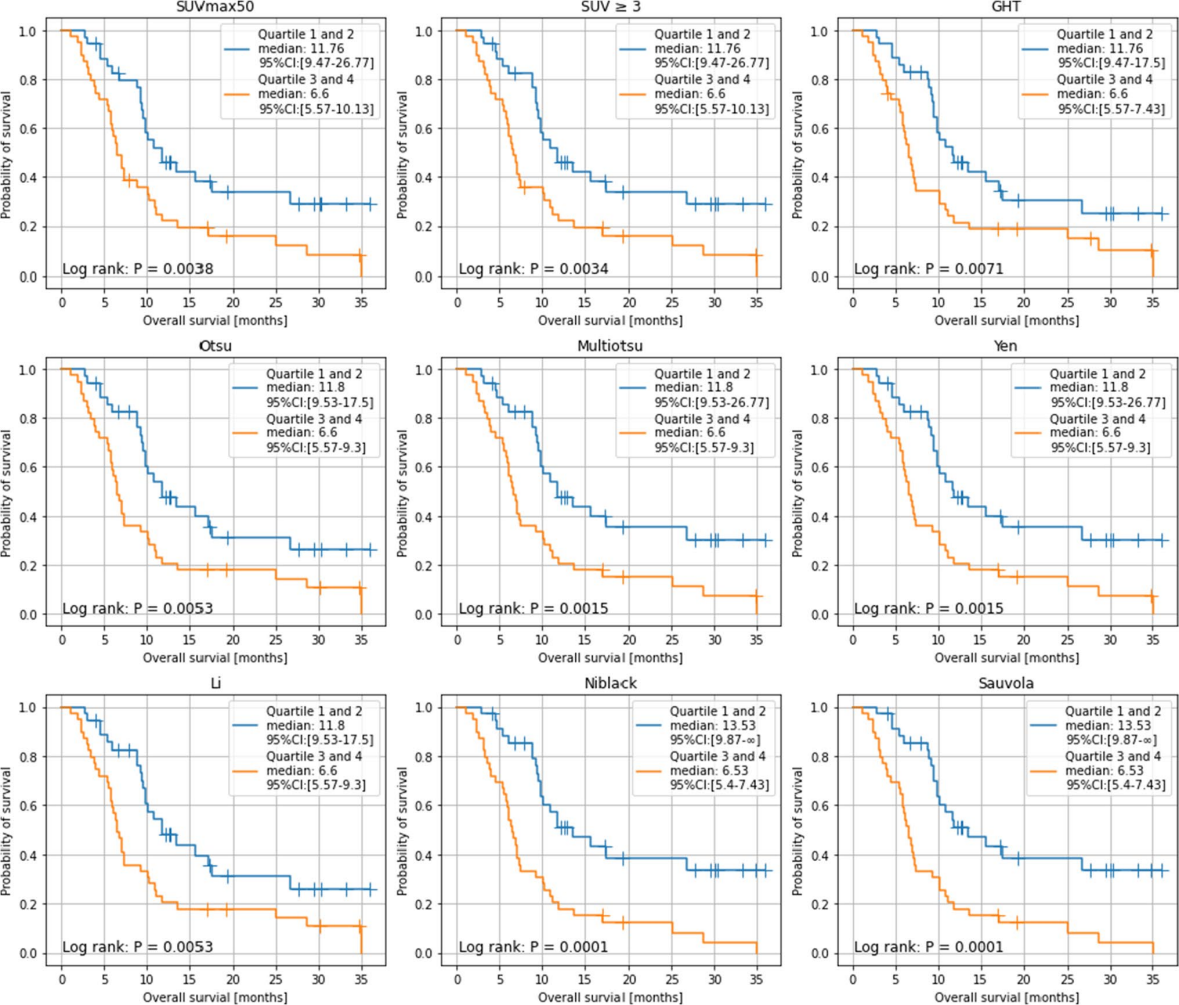
Kaplan–Meier analysis of OS in 78 patients with advanced prostate cancer undergoing [^177^Lu]Lu-PSMA-617 RLT. Comparison of OS stratified by whole-body molecular tumor volume for applied thresholding methods; cut-off at quartiles 1 and 2 vs. quartiles 3 and 4

The median survival time of the entire cohort was 9.53 months (95% CI [9.26 to 9.87]). A subgroup analysis was conducted only with patients followed until death due to the high number of patients lost to follow-up (22 out of 78 patients). Using these patients, the median survival time was 7.07 months (95% CI [6.6 to 7.43]). Stratification of patients into two groups along median whole-body PSMA-TV resulted in median survival time from 11.76 to 13.53 months for the patient group with lower tumor burden and 6.53 to 6.6 months for the patient group with higher tumor burden. The patient group with lower tumor burden had significantly higher probability of survival (*p* < 0.00625) in eight out of nine thresholding methods (Fig. 4); those methods were SUVmax50 (*p* = 0.0038), SUV ≥3 (*p* = 0.0034), Multiotsu (*p* = 0.0015), Yen (*p* = 0.0015), Niblack (*p* = 0.001), Sauvola (*p* = 0.0001), Otsu (*p* = 0.0053), and Li thresholding (*p* = 0.0053).

**Fig. 5:**
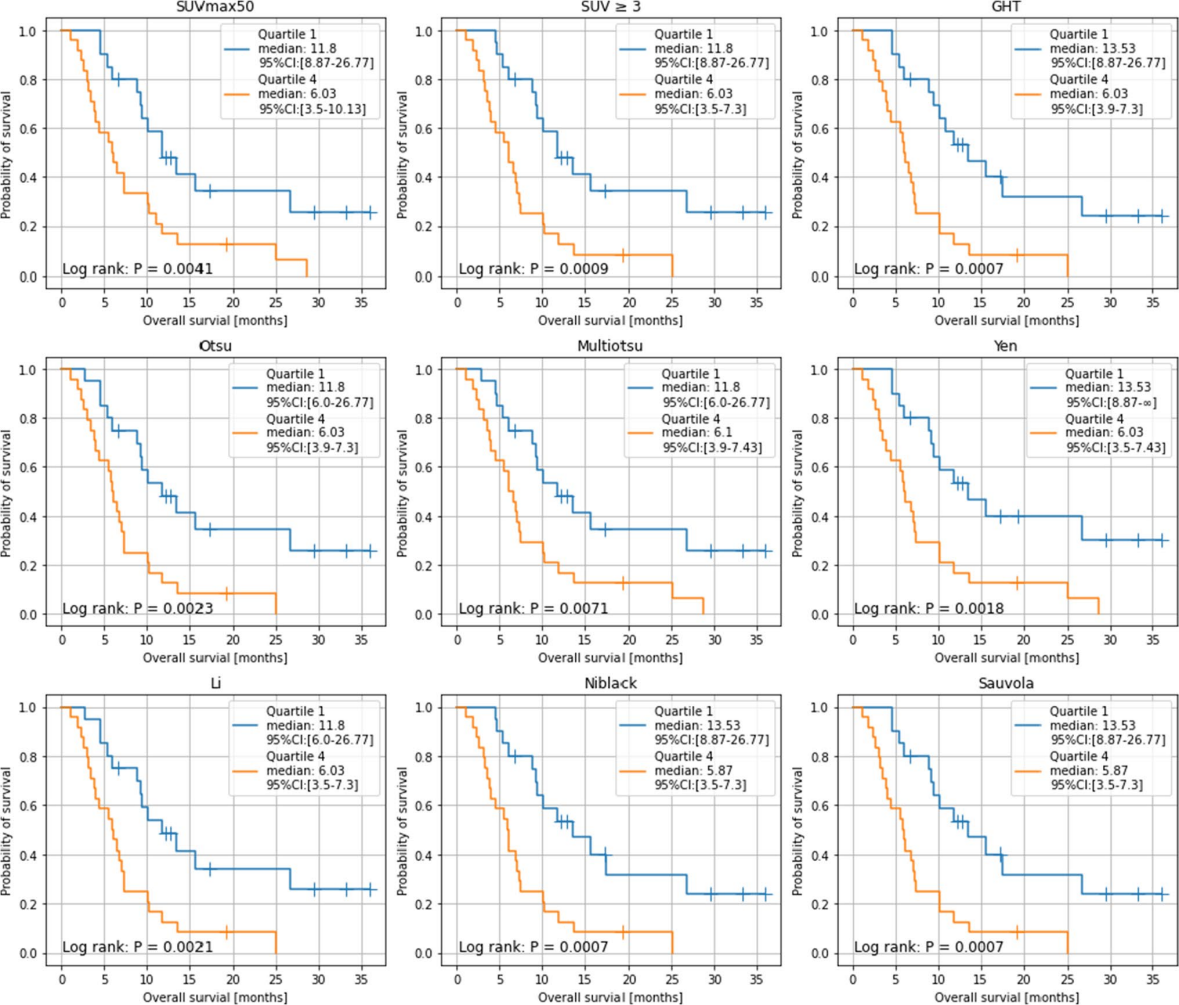
Kaplan–Meier analysis of OS in 78 patients with advanced prostate cancer undergoing [^177^Lu]Lu-PSMA-617 RLT. Comparison of OS stratified by whole-body molecular tumor volume for applied thresholding methods; cut-off at quartiles 1 vs. quartiles 4

When PSMA-TV was stratified at the first quartile versus the fourth quartile, stratified patient groups in all nine thresholding methods showed a significantly different probability of survival (*p* < 0.00625) (Fig. 5). In both stratifications, the best result was achieved by Niblack and Sauvola thresholding in terms of the *p* value (*p* = 0.0001 and *p* = 0.0007) and difference in median survival (13.53, 95% CI [9.87 to not reached] and 6.53, 95% CI [5.4 to 7.43]).

The whole-body PSMA-TVs of different thresholding methods were positive predictors of survival with a hazard ratio (HR) marginally above 1. The assumption of proportional hazards was largely met with the largest *p* value slightly above 0.05 (=0.0555). The HRs of different thresholding methods were in ascending order: GHT (1.0009, *p* = 0.0008), Niblack (1.001, *p* = 0.0008), Li and SUV ≥3 (HR = 1.0013, *p* = 0.0024 and *p* = 0.0008), Sauvola (HR = 1.0014, *p* = 0.0008), Otsu (HR = 1.0017, *p* = 0.0024), Yen (HR = 1.0022, *p* = 0.0016), Multiotsu (HR = 1.0033, *p* = 0.0024), and SUVmax50 (HR = 1.0035, *p* = 0.0008) (Table 3).

**Table 3:** Multivariate Cox regressions of overall survival for each thresholding method

Thresholding	HR	95% CI	*p* value
SUVmax50	1.0035	1.0014–1.0054	0.0008
SUV ≥3	1.0013	1.0007–1.0019	0.0008
GHT	1.0009	1.0004–1.0013	0.0008
Otsu	1.0017	1.0008–1.0027	0.0024
Multiotsu	1.0033	1.0015–1.0052	0.0024
Yen	1.0022	1.0010–1.0032	0.0016
Li	1.0013	1.0006–1.0021	0.0024
Niblack	1.0010	1.0005–1.0015	0.0008
Sauvola	1.0014	1.0005–1.0015	0.0008

### Validation of thresholding methods on phantom scan

**Fig. 6:**
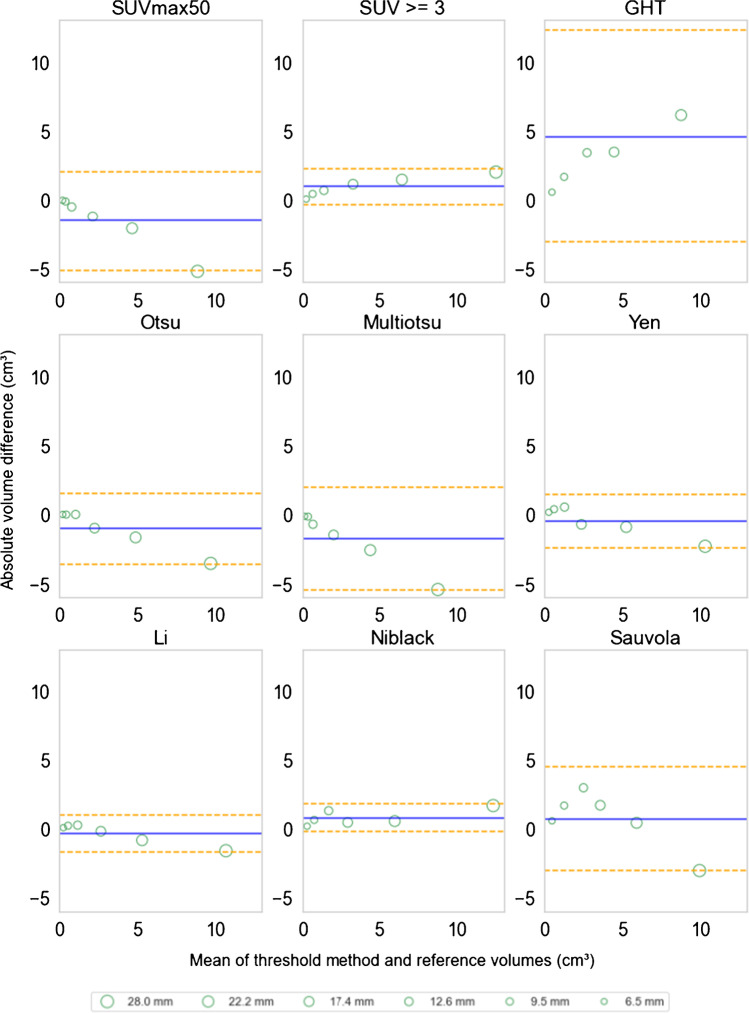
Bland–Altman plots comparing the thresholding methods for estimating spherical insert volumes in phantom scans. The central blue lines indicate the mean differences, and the orange dashed lines indicate the limits of agreements. The scatter plots are drawn as green circles with varying sizes. The measurements from PET images that were reconstructed with varying acquisition times were averaged for each spherical insert to better illustrate the diameter differences and their effect on volume estimations. The legend lists the inner diameter of spherical inserts in millimeters

For phantom scans, Li, Niblack, Yen, and SUV ≥3 methods yielded the best results with the lowest mean volume differences from the calculated spherical volumes of the phantom inserts (Fig. 6). The absolute mean volume differences were in ascending order (mean ± SD): Li (0.54 ± 0.58 cm^3^), Niblack (0.8 ± 0.55 cm^3^), Yen (0.85 ± 0.77 cm^3^), SUV ≥3 (0.97 ± 0.7 cm^3^), Otsu (1.06 ± 1.4 cm^3^), SUVmax50 (1.55 ± 1.97 cm^3^), Sauvola (1.73 ± 1.11 cm^3^), Multiotsu (1.76 ± 2.04 cm^3^), and GHT (4.59 ± 4.2 cm^3^). Noticeably, the SUV ≥3 method seemed to slightly underestimate smaller spherical volumes and overestimate larger spherical volumes, while the SUVmax50 thresholding behaved inversely.

## Discussion

In this study, we systematically investigated the effects of different thresholding algorithms on the quantification of whole-body molecular tumor volume in [^68^Ga]Ga-PSMA-11-PET of patients with advanced prostate cancer undergoing [^177^Lu]Lu-PSMA-617 radioligand therapy.

We demonstrate that several thresholding methods from computer vision are promising tools for quantifying whole-body PSMA-TV. The applied thresholding methods in this study correlate highly with currently applied thresholding methods and significantly predict the OS of patients with advanced prostate cancer.

The survival analysis with stratified whole-body PSMA-TV of different thresholding methods shows that particularly LAT methods (Niblack and Sauvola) yield better stratification of patients in risk groups with lower and higher tumor burdens than the baseline methods. In phantom scan re-evaluation, Niblack thresholding achieve higher accuracy in the PSMA-TV quantification of standardized, spherical, homogeneous lesions compared to established methods.

The novel aspect of the study is introducing thresholding computer vision techniques previously validated on generic image segmentation tasks, now for quantifying whole-body molecular tumor volume of patients with advanced prostate cancer. To date, several studies investigating whole-body molecular tumor volume have employed varying, liver-specific thresholding methods for segmentation tasks and PBT or fixed thresholding for volume quantification [9, 12]. The use of PBT is consistent with EANM guidelines for [^18^F]F-FDG-PET, which recommend the use of PBT of SUVmax on the delineated tumor foci for assessment of the molecular tumor volume [13]. The advantage of using PBT is well examined. Technically, PBT leads to smaller separated lesions, which can be better assigned to separate adjacent metastasis or automatically assigned to anatomical structures. The assignment to specific anatomical structures can be used, for example, for automatic exclusion of physiological tracer accumulations such as in the urinary tract [12, 20]. In this study, we could confirm that the whole-body PSMA-TVs calculated using PBT were altogether lower compared to the other applied thresholding methods, with the exception of Multiotsu (Fig. 2: higher tumor volume compared to baseline method led to a flatter course of the linear correlation). Nevertheless, PSMA-TV estimated using several thresholding methods (SUVmax50, Multiotsu, Yen, Niblack, and Sauvola thresholding) could significantly predict OS. In this regard, it was not feasible to calculate a global cut-off value for whole-body PSMA-TV for OS as the different applied methods resulted in widely varying PSMA-TV.

Recent studies also quantify the whole-body PSMA-TV as the sum of all PSMA-avid lesions with a fixed threshold value of SUV ≥3 with manual and semiautomatic exclusion of physiological uptake sites [10, 28–30]. While the use of global fixed threshold values was known to be suboptimal from the beginning, it is widely used because of the easy clinical accessibility without requiring additional computation [18, 30]. As computational tools continue to find their way into the clinical routine, we argue that an algorithm-driven thresholding strategy is less arbitrary and less prone to biases than thresholding with predefined percentage-based or fixed values. It would also better account for varying factors of scan procedures and individual differences between patients (e.g., metabolism, physiological uptake, time from injection to scan).

The widely divergent performance of HBT methods seems to indicate the presence of a multimodal histogram in which pathologic tracer uptake is concealed between the diffuse background noise and the high physiologic tracer accumulation in the urinary tract. Still, HBT methods could be valuable for segmentation tasks when combined with techniques that reliably eliminate physiological accumulations. In the past, HBT methods were neglected in the quantification of molecular volumes due to their susceptibility to partial volume effect and image blur, and concomitant overestimation of the molecular volumes [31, 32]. In this study, we could confirm this statement with the mean PSMA-TV of HBT methods systematically being higher than the quantified values of the baseline methods. In the phantom study, with human movement absent and reduced image blur, the global threshold methods exhibit rather an underestimation of the PSMA-TV. Nevertheless, the whole-body PSMA-TVs estimated by HBT methods still demonstrated a high correlation to the baseline methods and were applicable as a predictor of survival in patients with prostate cancer. In the prospect of clinical application as an imaging biomarker for the assessment of therapy response or prognosis of OS of patients with advanced prostate cancer, it could be argued that systematic overestimation or underestimation plays a minor role as long as the estimated whole-body PSMA-TV is inherently consistent within the applied algorithms.

The present study suffers from the limitations of retrospective study design and data analysis from a single site. The OS in our cohort was considerably lower with a median of 9.5 months than the reported OS in international multisite studies, with a median of 12.9 [33] and 15.3 months [17], respectively. The lower-than-expected OS was not due to the high number of lost to follow-up cases, which was examined in a subgroup with cases that have been followed until death. It was more likely due to very advanced metastatic castration-resistant prostate cancer cases in the cohort, as no exclusion criteria regarding an Eastern Cooperative Oncology Group performance status or adequate organ functions were specified in the retrospective design.

In some cases, the thresholding algorithms ultimately failed to delineate the tumor foci correctly, mainly when multiple liver metastases were present. Although the erroneous detection of tumor foci was visually identified in the binary mask overlayed maximum intensity projections (Fig. 1), the results were kept in the calculation to reduce the possibility of selection bias. This decision was in line with the study’s goal to explore and systematically evaluate the potential of different thresholding methods rather than to develop one best-suited method for quantifying the PSMA-TV in the evaluated patient cohort. The performance of each thresholding method also depends on the initialization parameters, which we did not optimize. Therefore, threshold methods with less promising results may perform better in different settings.

In future studies, histogram-based, local adaptive, and patient-specific thresholding methods could be combined to further automate and standardize the quantification method of the whole-body PSMA-TV. The thresholding methods could also efficiently run on reporting workstations in clinical practice as their computational effort is low, and no special hardware is needed. Moreover, the proposed thresholding algorithms are fully transparent, in contrast to some modern artificial intelligence algorithms with black-box characteristics that lack explainability of the underlying mechanisms [34]. In summary, our results demonstrate the potential of thresholding techniques from computer vision, which may help decrease bias, improve consistency, and help with agreement upon a more neutral standardized method. The results again demonstrate the great potential of PSMA-TV as an imaging biomarker for survival prognostication of patients with advanced prostate cancer.

## Conclusion

Several thresholding methods from computer vision are promising tools for semiautomatic quantification of whole-body PSMA-TV as an imaging biomarker for [^68^Ga]Ga-PSMA-11-PET. The proposed algorithm-driven thresholding strategy is less arbitrary and less prone to biases than thresholding with variations of predefined fixed or percentage values and can potentially improve the application of whole-body PSMA-TV as an imaging biomarker.

## Data Availability

The datasets generated during and/or analyzed during the current study are available from the corresponding author on reasonable request.

## Abbreviations

CI: Confidence interval
CT: Computed tomography
EANM: European Association of Nuclear Medicine
FDG: Fluordesoxyglucose
Ga: Gallium
GHT: Generalized histogram thresholding
HBT: Histogram-based thresholding
HR: Hazard ratio
IQR: Interquartile range
LAT: Local adaptive thresholding
MIWBAS: MI Whole Body Analysis Suite
OS: Overall survival
PET: Positron emission tomography
PSA: Prostate-specific antigen
PSMA: Prostate-specific membrane antigen
PSMA-TV: Prostate-specific membrane antigen tumor volume
PBT: Percentage-based thresholding
Q1: First quartile (25%)
Q3: Third quartile (75%)
RLT: Radioligand therapy
SD: Standard deviation
SUV: Standardized uptake value
SUVmax: Maximum standardized uptake value
SUVmax50: Threshold at 50% of the maximum standardized uptake value
UICC: Union for International Cancer Control
